# A link between damaging behaviour in pigs, sanitary conditions, and dietary protein and amino acid supply

**DOI:** 10.1371/journal.pone.0174688

**Published:** 2017-05-08

**Authors:** Yvonne van der Meer, Walter J. J. Gerrits, Alfons J. M. Jansman, Bas Kemp, J. Elizabeth Bolhuis

**Affiliations:** 1 Wageningen University, Department of Animal Sciences, Animal Nutrition Group, Wageningen, The Netherlands; 2 De Heus Animal Nutrition, Ede, The Netherlands; 3 Wageningen UR Livestock Research, Animal Nutrition, Wageningen, The Netherlands; 4 Wageningen University, Department of Animal Sciences, Adaptation Physiology Group, Wageningen, The Netherlands; INIA, SPAIN

## Abstract

The tendency to reduce crude protein (**CP**) levels in pig diets to increase protein efficiency may increase the occurrence of damaging behaviours such as ear and tail biting, particularly for pigs kept under suboptimal health conditions. We studied, in a 2×2×2 factorial design, 576 tail-docked growing-finishing entire male pigs in 64 pens, subjected to low (**LSC**) vs. high sanitary conditions (**HSC**), and fed a normal CP (**NP**) vs. a low CP diet (**LP**, 80% of NP) ad libitum, with a basal amino acid (**AA**) profile or supplemented AA profile with extra threonine, tryptophan and methionine. The HSC pigs were vaccinated in the first nine weeks of life and received antibiotics at arrival at experimental farm at ten weeks, after which they were kept in a disinfected part of the farm with a strict hygiene protocol. The LSC pigs were kept on the same farm in non-disinfected pens to which manure from another pig farm was introduced fortnightly. At 15, 18, and 24 weeks of age, prevalence of tail and ear damage and of tail and ear wounds was scored. At 20 and 23 weeks of age, frequencies of biting behaviour and aggression were scored for 10×10 min per pen per week. The prevalence of ear damage during the finisher phase (47 vs. 32% of pigs, *P* < 0.0001) and the frequency of ear biting (1.3 vs. 1.2 times per hour, *P* = 0.03) were increased in LSC compared with HSC pigs. This effect on ear biting was diet dependent, however, the supplemented AA profile reduced ear biting only in LSC pigs by 18% (SC × AA profile, *P* < 0.01). The prevalence of tail wounds was lower for pigs in LSC (13 ± 0.02) than for pigs in HSC (0.22 ± 0.03) in the grower phase (*P* < 0.007). Regardless of AA profile or sanitary status, LP pigs showed more ear biting (+20%, *P* < 0.05), tail biting (+25%, *P* < 0.10), belly nosing (+152%, *P* < 0.01), other oral manipulation directed at pen mates (+13%, *P* < 0.05), and aggression (+30%, *P* < 0.01) than NP pigs, with no effect on ear or tail damage. In conclusion, both low sanitary conditions and a reduction of dietary protein increase the occurrence of damaging behaviours in pigs and therefore may negatively impact pig welfare. Attention should be paid to the impact of dietary nutrient composition on pig behaviour and welfare, particularly when pigs are kept under suboptimal (sanitary) conditions.

## Introduction

In the pig industry, reduction of dietary protein contributes to the goal of making livestock farming more nutrient efficient. Reducing dietary protein level seems possible without compromising the growth performance of pigs if supplementary dietary essential amino acids **(AA)** are provided [1, 2]. It has been suggested, however, that feeding diets with low dietary protein levels may increase the occurrence of damaging behaviours such as ear and tail biting [3, 4]. If particular nutrients are limiting for growth or immune functioning, pigs might increase their foraging behaviour and alter their feed preferences to satisfy their nutritional needs [4]. Indeed, pigs provided a low-protein diet, although fed ad libitum, showed more foraging activities than pigs fed a diet with adequate protein levels [4]. Pigs may, in turn, redirect their natural foraging and exploratory behaviour to pen mates, particularly when suitable rooting substrates are not or only sparsely available. The nosing, chewing, rooting and sucking directed at the tails, ears and other body parts of conspecifics may culminate in vigorous biting and, as a consequence might lead to wounds [5, 6]. Once wounds have developed, biting behaviour may escalate, particularly in pigs fed low-protein diets, which are even more attracted to blood [7].

Dietary AA imbalances have also been associated with injurious biting behaviour. Several studies suggest that pigs respond to a shortage of specific essential AA, including methionine (**Met**), threonine (**Thr**), and tryptophan (**Trp**), by adjusting their feed selection behaviour accordingly [8–11]. Due to a relative shortage in AA, foraging behaviour and redirected biting behaviour might increase, and blood may become more attractive. In line with the latter, McIntyre and Edwards [12] found that the preference to chew on a blood-soaked rope as opposed to a water-soaked rope increased when pigs were fed a diet low in Trp. The increased preference for blood in pigs fed low-Trp diets may, consecutively, influence injurious biting. In support of this, weanling pigs fed diets supplemented with free Trp in excess to requirement bit less on the tails and ears of their pen mates [13].

Dietary protein deficiencies or AA imbalances may influence behaviour through their effects on brain neurotransmitters, as many of these are synthesized from particular AA. The effects of dietary Trp concentration, for instance, on aggressive behaviour and stress as reported in numerous species [14–18] have been linked to its role as a precursor of serotonin (5-hydroxytryptamine; **5-HT**). Recent studies have provided evidence for a link between damaging biting behaviour and 5-HT. Pigs had lowered blood platelet 5-HT storage and higher blood platelet 5-HT uptake velocities in phases of life during which they were classified as tail biters [19]. Additionally, Valros et al. [20] reported that the Trp and central 5-HT metabolism of tail biters was different from that of victims and non-biters.

Apart from dietary influences on injurious biting behaviour, a poor health status has been identified as one of the risk factors for this multifactorial problem [21, 22]. Respiratory disease and tail biting for example, seem to be associated at animal level [23] and at farm level [24]. The exact nature of the relationship between health and biting behaviour is not completely understood. On the one hand, a low health status or poor sanitary conditions could lead to increased requirements for specific nutrients, particularly those involved in immune system activation, such as Trp, and in this way contribute to biting behaviour. On the other hand, biting can also cause health problems, as it frequently leads to inflammatory responses [25] as supported by Heinonen et al. [26] who found higher acute phase concentrations in blood in tail bitten pigs compared with non tail-bitten control pigs. Finally, poor health and injurious biting could also partly reflect suboptimal management, feed or climatic conditions, all of which are known to exacerbate both health and behavioural problems [24, 27, 28], without necessarily being causally related. Cause and effect are thus difficult to disentangle in the health-biting relationships reported so far, and, to the best of our knowledge, no studies have been executed on the topic in which health status was experimentally manipulated rather than only assessed.

Reducing dietary protein concentrations on pig farms with poor health conditions may therefore aggravate behavioural problems. Therefore we studied the combined effects of diverging sanitary conditions, dietary protein level and AA profile on redirected biting behaviours and tail and ear damage of pigs. Performance and health parameters of the pigs in the present study have been published elsewhere [29].

## Animals. materials and methods

The experiment was approved by the Animal Care and Use Committee of Wageningen University.

### Experimental design

A 2 × 2 × 2 factorial design was applied with sanitary conditions (high: HSC, or low: LSC), dietary crude protein level (low: LP, or normal: NP), and dietary AA profile (basal: **AA-B** or supplemented: **AA-S**) as experimental factors at pen level.

### Animals and treatments

In total 576, entire male tail-docked Topigs 20 × Tempo (Topigs, Helvoirt, The Netherlands) piglets were selected on a commercial farm. Half of the piglets involved were subjected to the LSC and the other half to the HSC treatment. The piglets for both LSC and HSC were selected from the same farrowing rooms and were allocated per litter; all boar piglets of a sow were selected for either LSC or HSC. After weaning (mean age 24 d), LSC and HSC pigs were group-housed in different rooms to prevent cross-vaccination. The HSC piglets, and not LSC piglets, were vaccinated against *Mycoplasma hyopneumoniae*, porcine circovirus type 2, porcine reproductive and respiratory syndrome, *Lawsonia intracellularis*, *Actinobacillus pleuropneumoniae*, and influenza A virus in the first nine weeks of age as specified in Van der Meer et al. [29].

The HSC and LSC pigs were transported separately to another commercial farm where the experiment was conducted (Vlierbos V.O.F., Neerloon, The Netherlands) with half of the pigs at 10 weeks of age and the other half at 11 weeks of age. (two batches of 288; LSC: 144, HSC: 144).

Upon arrival, pigs were allocated to their pen based on body weight (**BW**) in order to minimize variation in BW between pens (mean BW ± standard error of the mean; LSC batch 1: 17.3 ± 0.06 kg, LSC batch 2: 18.1 ± 0.07 kg, HSC batch 1: 15.9 ± 0.07 kg, HSC batch 2: 17.4 ± 0.07 kg). In total 64 concrete pens were used, each pen had a partly slatted floor and contained nine pigs (0.8 m^2^ space per pig). All pens were distributed over eight rooms; four rooms we selected for LSC pigs and four rooms for HSC pigs. Per room each experimental diet was distributed to two pens, resulting in one repetition of treatment combination per room. Distribution of experimental diet per room was done randomly. Per treatment combination there were eight replicates (pens). Each room had separate manure pits and separate negative pressure mechanical ventilation regulation. The temperature in the rooms was set at 24°C at start of the experiment and was decreased to 20°C during the experiment. The LSC rooms were not cleaned after containing a previous batch of commercial finisher pigs that left the facility two days before, and no specific hygiene protocol was applied to these rooms. Starting at five weeks after arrival, fresh manure of another commercial pig farm was spread in the LSC pens every two weeks until end of the experiment to enhance antigenic pressure. The LSC pigs did not receive any preventative medication. In contrast, HSC pigs received a dose of antibiotics (Fenflor; AUV Veterinary Services B.V., Cuijk, the Netherlands; 1 mL/pig, intramuscular at Day 1 and 3 of the experiment) after arrival at 10 weeks of age and were placed in four disinfected rooms in a distinct part of the pig facility with a strict hygiene protocol (see [29]).

Animals were monitored for the complete fattening period, divided in three phases, i.e. starter (0–34 d), grower (35–49 d), and finisher phase (from day 50 until a target average pen BW of 110 kg).

### Diets, feeding and analysis

Pens were allocated to a diet with either a low crude protein (CP) level (LP) or a normal CP level (NP), each having either a basal AA-profile or a supplemented AA-profile, resulting in four different diets: LP-AA-B, LP-AA-S, NP-AA-B, NP-AA-S.

The apparent ileal digestible (AID) Lys/ MJ NE ratio of the diets was reduced in each subsequent phase of the experiment to follow a three phase feeding system. For the NP diet, the Lys/ MJ NE ratio was set to 95% of the requirement values for boars, as published by NRC [30], to prevent dietary energy to be limiting for growth performance. The respective diets contained 0.90 g AID Lys/ MJ NE for the starter phase, 0.81 g AID Lys/ MJ NE for the grower phase, and 0.75 g AID Lys/ MJ NE for the finisher phase. The LP diets were created by decreasing the inclusion level of all protein-containing ingredients by 20% in exchange for maize starch and cellulose, resulting in diets with 0.72 g AID Lys/ MJ NE for the starter phase, 0.65 g AID Lys/ MJ NE for the grower phase, and 0.60 g AID Lys/NE for the finisher phase.

The basal AA-profile (AA-B) was designed to cover the AA requirements for body protein deposition [30–32] and to cover AA losses associated with basal endogenous proteins in ileal digesta [30, 33], AA losses in skin and hair [30], and AA losses related to cell and tissue turnover in the body [34], as specified in [29]). The supplemented AA-profile (AA-S) was derived from the AA-B profile by increasing the Met, Thr, and Trp ratio relative to Lys on a AID basis by 20%. These AA are believed to be increasingly important as building blocks for synthesis of specific proteins, such as acute phase proteins, synthesized in case of immune system activation [35–38], and effects on immune processes [39]. The composition of the diets is shown in Tables 1–3, and the AA-profiles are shown in data in S1 File. The calculated NE value in Tables 1–3 was based on the Centraal Veevoeder Bureau livestock feed table [40]. All diets were isocaloric on a NE basis and provided as pellets during the experiment. Per pen one single space feeder and one nipple drinker were present and feed and water were offered *ad libitum*.

**Table 1:** Ingredient and nutrient composition of the starter diets.

		LP^1^		NP^1^	
Item		AA-B^2^	AA-S^2^	AA-B	AA-S
Ingredient, g/kg of feed					
	Maize	320.00	320.00	400.00	400.00
	Soybean meal	182.02	182.00	227.54	227.54
	Barley	160.00	160.00	200.00	200.00
	Wheat	45.53	45.53	56.91	56.91
	Maize starch	206.79	204.64	40.65	37.90
	Sugarcane molasses	20.00	20.00	20.00	20.00
	Limestone	13.94	13.94	14.11	14.11
	Monocalcium phosphate	9.99	9.99	8.93	8.93
	Soybean oil	10.65	10.98	15.98	16.38
	Vitamin + mineral mix^3^	5.00	5.00	5.00	5.00
	Salt	3.19	3.19	3.83	3.83
	L-lysine HCl	1.95	1.94	2.35	2.35
	Titanium dioxide	2.50	2.50	2.50	2.50
	Sodium bicarbonate	2.58	2.58	1.34	1.34
	L-threonine	0.60	1.46	0.63	1.71
	L-tryptophan	0.03	0.31	0.00	0.37
	DL-methionine	0.23	0.94	0.23	1.13
	Cellulose^4^	15.00	15.00	0.00	0.00
Nutrients calculated, g/kg					
	NE, MJ/kg^5^	9.80	9.80	9.80	9.80
	DM	889.6	889.8	893.2	884.9
	Crude protein	138.0	136.0	168.0	167.0
	Starch^4^	474.1	472.3	410.0	407.7
	Lys^6^	8.60	8.60	10.50	10.50
	Thr^6^	5.40	6.20	6.60	7.40
	Trp^6^	1.70	1.90	2.00	2.30
	Met + Cys^6^	4.30	4.80	5.20	5.90
	Ile^6^	5.60	5.60	6.90	6.80
	Arg^6^	8.30	8.40	10.50	10.30
	Phe^6^	6.60	6.60	8.20	8.10
	His^6^	3.40	3.40	4.20	4.20
	Leu^6^	10.9	11.00	13.60	13.40
	Tyr^6^	4.40	4.50	5.70	5.70
	Val^6^	6.40	6.40	7.90	7.70

^1^ LP = low CP concentration diet, NP = normal CP concentration diet.

^2^ AA-B = basal dietary AA profile, AA-S = supplemented dietary AA profile containing 20% more Met, Thr, and Trp compared with the basal profile.

^3^ Supplied the following per kilogram of diet: 3.0 mg riboflavin, 20 mg niacine, 20 mg d-pantothenic acid, 10 mg choline chloride, 0.015 mg cyanocobalamin, 40 mg dl-α-tocopheryl acetate, 1.5 mg menadione, 6,000 IU retinyl acetate, 1,200 IU cholecalciferol, 0.2 mg folic acid, 1.0 mg thiamin, 1.0 mg pyridoxine HCl, 50 mg manganese oxide, 267 mg iron SO_4_·H_2_O, 60 mg copper SO_4_·5H_2_O, 140 mg zinc SO_4_·H_2_O, 0.44 mg disodium selenium trioxide,1.0 mg potassium iodate.

^4^ Opticell (Agromed Austri GmbH, Kremsmünster, Austria).

^5^ Based on chemical composition, digestibility, and energy value for pigs from the Centraal Veevoeder Bureau livestock feed table.

^6^ Analyzed values.

**Table 2:** Ingredient and nutrient composition of the grower diets.

		LP^1^		NP^1^	
Item		AA-B^2^	AA-S^2^	AA-B	AA-S
Ingredient, g/kg of feed					
	Maize	400.00	400.00	500.00	500.00
	Soybean meal	138.15	138.15	172.69	172.69
	Barley	171.36	171.36	214.19	214.19
	Maize starch	199.19	197.24	43.34	40.86
	Wheat	20.00	20.00	20.00	20.00
	Sugarcane molasses	13.11	13.11	13.33	13.33
	Limestone	8.62	8.62	7.67	7.67
	Monocalcium phosphate	9.19	9.48	10.20	10.57
	Soybean oil	5.00	5.00	5.00	5.00
	Vitamin + mineral mix^3^	3.95	3.95	3.61	3.61
	Salt	2.45	2.45	2.92	2.92
	L-lysine HCl	2.50	2.50	2.50	2.50
	Titanium dioxide	1.56	1.56	2.00	2.00
	Sodium bicarbonate	3.87	3.87	1.52	1.52
	L-threonine	0.72	1.49	0.74	1.72
	L-tryptophan	0.21	0.46	0.22	0.55
	DL-methionine	0.12	0.76	0.07	0.87
	Cellulose^4^	20.00	20.00	0.00	0.00
Nutrients calculated, g/kg					
	NE, MJ/kg^5^	9.84	9.84	9.84	9.84
	DM	883.7	885.9	882.8	887.7
	Crude protein	124.0	124.0	152.0	152.0
	Starch^5^	497.1	495.5	448.9	446.9
	Lys^6^	8.00	7.90	9.70	10.00
	Thr^6^	5.10	5.90	5.90	7.00
	Trp^6^	1.56	1.75	1.91	2.16
	Met + Cys^6^	3.98	4.52	4.76	5.58
	Ile^6^	4.80	4.70	5.90	5.80
	Arg^6^	7.20	7.00	9.00	9.00
	Phe^6^	5.80	5.70	7.10	7.10
	His^6^	3.00	3.00	3.70	3.60
	Leu^6^	10.0	9.90	12.3	12.3
	Tyr^6^	3.80	3.90	5.00	5.00
	Val^6^	5.70	5.80	6.90	6.90

^1^ LP = low CP concentration diet, NP = normal CP concentration diet.

^2^ AA-B = basal dietary AA profile, AA-S = supplemented dietary AA profile containing 20% more Met, Thr, and Trp compared with the basal profile.

^3^ Supplied the following per kilopgram of diet: 3.0 mg riboflavin, 20 mg niacine, 20 mg d-pantothenic acid, 10 mg choline chloride, 0.015 mg cyanocobalamin, 40 mg dl-α-tocopheryl acetate, 1.5 mg menadione, 6,000 IU retinyl acetate, 1,200 IU cholecalciferol, 0.2 mg folic acid, 1.0 mg thiamin, 1.0 mg pyridoxine HCl, 50 mg manganese oxide, 267 mg iron SO_4_·H_2_O, 60 mg copper SO_4_·5H_2_O, 140 mg zinc SO_4_·H_2_O, 0.44 mg disodium selenium trioxide,1.0 mg potassium iodate.

^4^ Opticell (Agromed Austri GmbH, Kremsmünster, Austria).

^5^ Based on chemical composition, digestibility, and energy value for pigs from the Centraal Veevoeder Bureau livestock feed table.

^6^ Analyzed values.

**Table 3:** Ingredient and nutrient composition of the finisher diets.

		LP^1^		NP^1^	
Item		AA-B^2^	AA-S^2^	AA-B	AA-S
Ingredient, g/kg of feed					
	Maize	360.10	360.10	450.10	450.10
	Soybean meal	115.70	115.70	144.60	144.60
	Barley	240.00	240.00	300.00	300.00
	Maize starch	187.20	185.30	36.40	34.10
	Wheat	20.00	20.00	20.00	20.00
	Sugarcane molasses	12.20	12.20	12.50	12.50
	Limestone	7.40	7.40	6.50	6.50
	Monocalcium phosphate	14.00	14.30	13.20	13.60
	Soybean oil	5.00	5.00	5.00	5.00
	Vitamin + mineral mix^3^	2.30	2.30	2.60	2.60
	Salt	2.40	2.40	2.80	2.80
	L-lysine HCl	2.50	2.50	2.50	2.50
	Titanium dioxide	5.30	5.30	3.00	3.00
	Sodium bicarbonate	0.00	0.60	0.00	0.70
	L-threonine	0.70	1.40	0.70	1.60
	L-tryptophan	0.10	0.40	0.10	0.40
	DL-methionine	0.10	0.10	0.00	0.00
	Cellulose^4^	25.00	25.00	0.00	0.00
Nutrients calculated, g/kg					
	NE, MJ/kg^5^	9.84	9.84	9.84	9.84
	DM	885.9	886.5	881.6	887.7
	Crude protein	132.0	126.0	151.0	148.0
	Starch^5^	541.7	509.4	541.7	540.2
	Lys^6^	8.00	7.60	8.90	8.90
	Thr^6^	5.30	5.60	5.90	6.50
	Trp^6^	1.51	1.62	1.68	1.91
	Met + Cys^6^	4.03	4.22	4.68	5.04
	Ile^6^	4.90	4.60	5.70	5.60
	Arg^6^	7.30	6.80	8.20	8.20
	Phe^6^	6.00	5.60	6.80	6.80
	His^6^	3.10	2.90	3.50	3.50
	Leu^6^	10.10	9.60	11.90	11.30
	Tyr^6^	4.00	3.70	4.70	4.70
	Val^6^	6.00	5.70	6.60	6.60

^1^ LP = low CP concentration diet, NP = normal CP concentration diet.

^2^ AA-B = basal dietary AA profile, AA-S = supplemented dietary AA profile containing 20% more Met, Thr, and Trp compared with the basal profile.

^3^ Supplied the following per kilogram of diet: 3.0 mg riboflavin, 20 mg niacine, 20 mg d-pantothenic acid, 10 mg choline chloride, 0.015 mg cyanocobalamin, 40 mg dl-α-tocopheryl acetate, 1.5 mg menadione, 6,000 IU retinyl acetate, 1,200 IU cholecalciferol, 0.2 mg folic acid, 1.0 mg thiamin, 1.0 mg pyridoxine HCl, 50 mg manganese oxide, 267 mg iron SO_4_·H_2_O, 60 mg copper SO_4_·5H_2_O, 140 mg zinc SO_4_·H_2_O, 0.44 mg disodium selenium trioxide,1.0 mg potassium iodate.

^4^ Opticell (Agromed Austri GmbH, Kremsmünster, Austria).

^5^ Based on chemical composition, digestibility, and energy value for pigs from the Centraal Veevoeder Bureau livestock feed table.

^6^ Analyzed values.

Diets were analyzed for AA composition by acid hydrolysis at 110°C for 23 h and ion-exchange chromatography with post-column derivatization with ninhydrin [41] and Trp by alkaline hydrolysis at 110°C for 20 h ion-exchange chromatography with fluorescence detection [42].

### Behavioural observations

Pigs were observed at 20 (grower phase) and 23 (finisher phase) weeks of age using behaviour sampling. Frequencies of the behaviours, described in Table 4, were recorded during live observations from 8.00–12.20 h and 14.00–16.50 h during two consecutive days. In case of continuous biting for > 30 sec, after 30 sec a new occurrence was scored. Animals in each pen were observed 10 × 10 min, resulting in 100 min of observations per pen per week of observation. Distribution of the 10-min observation blocks over the day and over the two observation days was balanced for experimental treatments. Observers were blind to the dietary treatments and divided over the pens balanced for treatment. All four observers were trained by the same person before behavioural observations.

**Table 4:** Ethogram.

Behaviour	Description
**Oral manipulation of group mates**	
Tail biting	Nibbling, sucking or chewing the tail of a pen mate
Ear biting	Nibbling, sucking or chewing the ear of a pen mate
Manipulating other	Nibbling, sucking or chewing of another part of the body of a pen mate
**Aggression**	
Fighting	Ramming or pushing a pen mate with or without biting the other pen mate. Can be either mutual or unilateral
Fighting at feeder	Pushing, head knocking or biting a pen mate at the feeder
**Belly nosing**	Rubbing the belly of a pen mate with up and down snout movements
**Mounting**	Standing on hind legs while having front legs on other pig's body
**Enrichment object biting**	Chewing on an enrichment object provided in the pen. The enrichment object was a chain with a hard plastic pipe fixed at the top of the pen wall.

### Damage and wound scores

Tail damage was recorded at 15, 18 and 24 weeks of age as indicator of being tail bitten, using the following scores as described in Ursinus et al. [43]: 1. No tail damage, 2. Bite marks; thin scratches. The individual bite marks have the size of a pinhead, 3. Small wound; clearly visible lesion with fresh or dried blood on the (top of the) tail, but the tail retains its entire length, 4. Medium wound; clearly visible lesion with fresh or dried blood on the tail and the tail is partly shortened, 5. Severe wound; lesion with fresh or dried blood, the tail is completely removed. Ear damage was recorded together with the tail damage scoring. Only damage to the backside of the ears was recorded. Scoring was done as follows: 1. No ear damage, 2. Top or bottom lesions; thin scratches, 3. Top and bottom lesions; thin scratches, 4. Severe damage, part of ear is missing. These scores were determined as described by Ursinus et al. [43]. Cases of ear necrosis were given a score of 5. It is not fully known whether ear necrosis is a result of ear biting. Recent epidemiological studies point to a possible infection route for ear necrosis through biting, however, the spread of ear necrosis in pig herds is difficult to explain solely by opportunistic bacterial infections [44, 45]. Damage scores of both ears were recorded, and the average score of both ears was used for further analysis, resulting in one value per pig per recording week for further analysis. Pigs received a binary score for both tail and ear damage: (0) no damage (score1) vs. (1) damage (score >1) per phase. Similarly, pigs received a binary score for having (0) no wound (score < 3 for tails, and score < 4 for ears) vs. (1) a wound (score > 2 for tails, and > 3 for ears) per phase. These scores were averaged per pen and phase and used for further analysis. In case of a score > 4 for tail damage or ear damage, pigs were individually treated with a iodine spray and another wound spray to prevent bacterial infections (MS jodium bruin and MS protect verband spray, MS Schippers B.V., Bladel, The Netherlands). No pigs were removed from the experiment.

### Statistical analysis

Statistical analyses was performed using SAS (SAS 9.3, Institute Inc.). Behavioural frequencies were averaged per pen and phase (grower and finisher) before analysis. Residuals of the response variables were checked for normality, and if needed square root transformed. All variables for behavioural frequencies were analyzed in a Mixed Model that included sanitary condition, dietary CP level, dietary AA profile, batch, phase, sanitary condition × dietary CP level, and sanitary conditions × dietary AA profile as fixed effects. Other interactions were deleted from the model when not significant. The effect of room nested within sanitary conditions was used as a random effect to correct for differences between rooms.

To analyze damage scores, the prevalence of damage was analyzed by expressing damage as a 0–1 variable (0 = no damage, 1 = damage) per pig per phase. The pen-averaged prevalences were analyzed per phase by a Mixed model that included sanitary condition, dietary CP level, dietary AA profile, batch, sanitary condition × dietary CP level, and sanitary conditions × dietary AA profile as fixed effects. Other interactions were deleted from the model when not significant. The effect of room nested within sanitary condition was used as a random effect to correct for differences between rooms. The prevalence of wounds was analyzed by expressing wounds as a 0–1 variable (0 = no wound, 1 = wound) per pig per phase and expressed as prevalence per pen per phase. The pen-averaged wound prevalences were analyzed per phase with the same model as the pen averaged damage prevelances. All values are presented as raw means ± standard error of the mean.

## Results

### Behavioural observations

Oral manipulation of group mates occurred more often (4.1 ± 0.4 times per pig per hour) than chewing on enrichment object (1.0 ± 0.2) and aggression (2.4 ± 0.3). Oral manipulation was less directed at tails (0.3 ± 0.1) than ears (1.5 ± 0.2) or body parts other than tails or ears (2.3 ± 0.3). Frequency of belly nosing, was rather low (0.1 ± 0.1), and the same held for mounting (0.4 ± 0.1).

**Fig 1:**
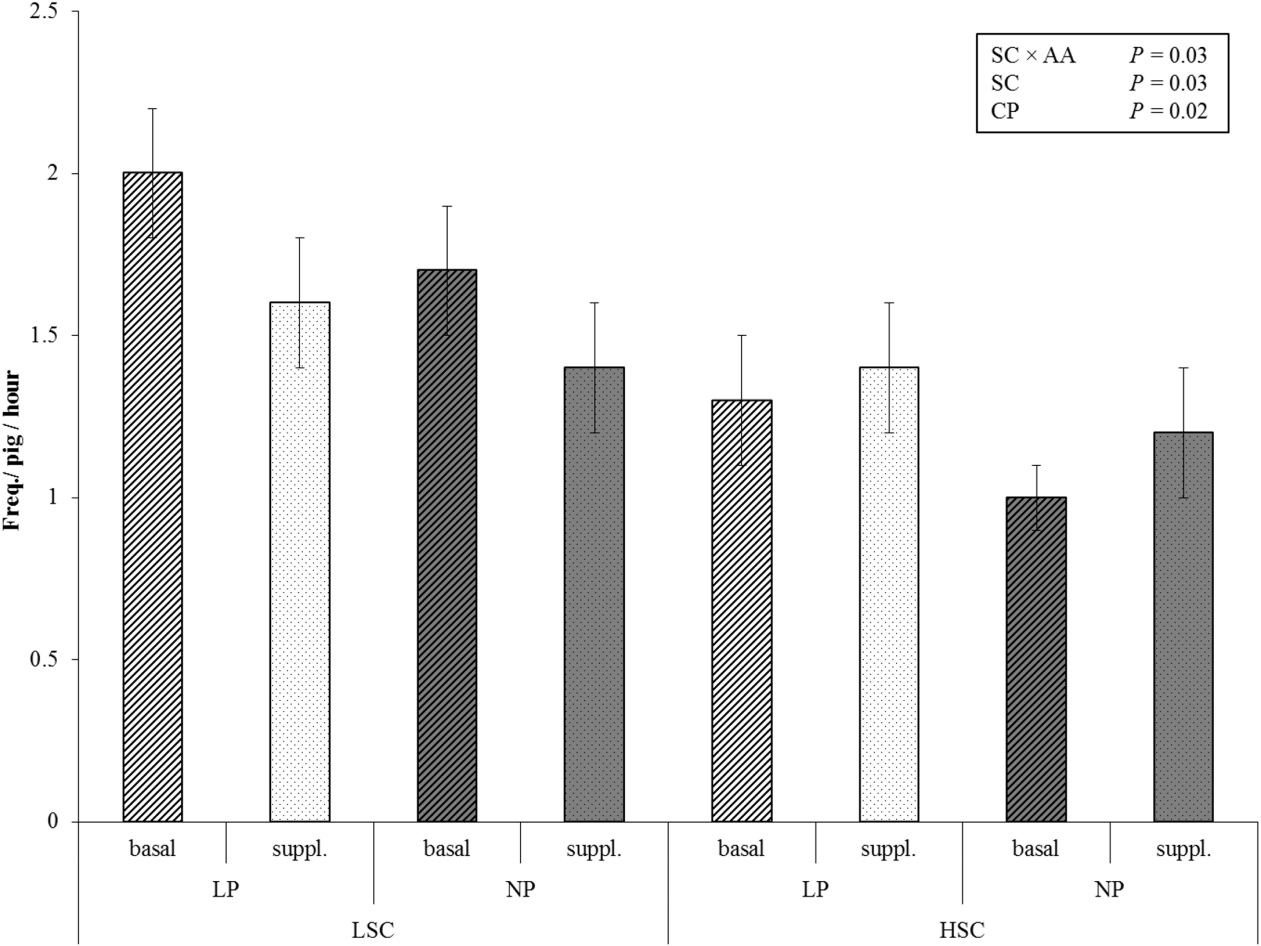
Frequency of ear biting in pigs kept under different sanitary conditions and fed a diet with either a low or normal dietary protein level and a basal or supplemented amino acid profile. Ear biting was recorded during live ad libitum sampling for 100 min per pen in total. The bars represent the raw means per treatment group ± standard error of the mean. LSC = low sanitary conditions, HSC = high sanitary conditions, LP = low dietary crude protein level (white bars). NP = normal dietary crude protein level (grey bars), AA-B = basal dietary amino acid profile (striped bars), AA-S = supplemented dietary amino acid profile containing 20% more Met, Thr, and Trp compared with the basal profile (dotted bars), SC = sanitary conditions, AA = amino acid profile.

Frequency of total oral manipulation of pen mates (chewing or nibbling tail, ear or other body parts) was higher for LSC than for HSC pigs (4.5 vs. 3.8 times per pig per hour, *P* ≤ 0.05, Table 5). Ear biting was affected by the interaction between sanitary conditions and dietary AA treatment (*P* ≤ 0.05, Fig 1). Ear biting was more frequently recorded for LSC pigs fed AA-B diets compared with LSC pigs fed AA-S diets (1.9 vs. 1.5 times per hour), whereas, frequency of ear biting in HSC pigs was similar for pigs fed the AA-B diet and the AA-S diets (1.2 vs. 1.3 times per hour). For tail biting behaviour also an interaction between sanitary conditions and dietary AA level was found (*P* ≤ 0.05). Tail biting was more frequently recorded in HSC pigs fed AA-B diets compared with HSC pigs fed AA-S diets (0.4 ± 0.1 vs. 0.3 ± 0.1 times per hour), whereas dietary AA level did not affect frequency of tail biting in LSC pigs (0.4 ± 0.1 vs. 0.4 ± 0.1 times per hour). Sanitary conditions, dietary AA profile or their interaction did not affect chewing on an enrichment object or aggressive behaviour.

**Table 5:** Frequencies of behaviours collected by behaviour sampling during the grower and finisher phase in pigs kept under low or high sanitary conditions, and provided with diets containing either low or normal protein levels and either a basal or a supplemented amino acid profile.

	LSC^2^							HSC^2^												
	LP^3^				NP^3^			LP				NP			*p*-values^5^					
Behaviour^1^	AA-B^4^		AA-S^4^		AA-B		AA-S	AA-B		AA-S		AA-B		AA-S	SC	CP	AA	batch	SC × CP	SC × AA
n	8		8		8		8	8		8		8		8						
**Oral manipulation of group mates**	5.0 ± 0.4		4.5 ± 0.4		4.3 ± 0.4		4.2 ± 0.5	4.3 ± 0.3		4.0 ± 0.3		3.5 ± 0.4		3.3 ± 0.4	**0.02**	**0.002**	0.23	**0.0004**	0.54	0.89
Tail biting	0.4 ± 0.1		0.4 ± 0.1		0.3 ± 0.0		0.3 ± 0.1	0.4 ± 0.0		0.3 ± 0.1		0.4 ± 0.1		0.2 ± 0.0	0.59	0.06	0.23	0.09	0.58	**0.02**
Ear biting	2.0 ± 0.2		1.6 ± 0.2		1.7 ± 0.2		1.4 ± 0.2	1.3 ± 0.2		1.4 ± 0.2		1.0 ± 0.1		1.2 ± 0.2	**0.03**	**0.02**	0.38	0.95	0.99	**0.03**
Manipulating other	2.6 ± 0.3		2.5 ± 0.3		2.3 ± 0.2		2.4 ± 0.3	2.6 ± 0.2		2.3 ± 0.2		2.1 ± 0.3		1.9 ± 0.2	0.19	0.04	0.47	**< .0001**	0.30	0.41
** **																				
**Chewing enrichment object**	1.3 ± 0.2		1.3 ± 0.3		0.8 ± 0.2		0.8 ± 0.2	1.0 ± 0.2		1.2 ± 0.2		0.8 ± 0.2		0.6 ± 0.2	0.54	**0.002**	0.99	0.48	0.59	0.87
** **																				
**Aggression**	2.8 ± 0.3		2.8 ± 0.3		2.3 ± 0.3		2.1 ± 0.2	2.7 ± 0.3		2.3 ± 0.3		2.2 ± 0.3		1.6 ± 0.3	0.37	**0.001**	0.14	0.65	0.95	0.29
Fighting	1.4 ± 0.2		1.2 ± 0.2		0.8 ± 0.1		0.9 ± 0.2	1.4 ± 0.2		1.3 ± 0.2		1.2 ± 0.2		0.7 ± 0.1	0.84	**0.001**	0.17	0.35	0.80	0.22
Feeder fighting	1.4 ± 0.2		1.6 ± 0.2		1.5 ± 0.2		1.2 ± 0.2	1.3 ± 0.2		1.1 ± 0.1		1.0 ± 0.2		0.9 ± 0.2	0.25	0.11	0.41	0.23	0.72	0.74
** **																			** **	
**Belly nosing**	0.2 ± 0.1		0.3 ± 0.1		0.1 ± 0.0		0.1 ± 0.0	0.1 ± 0.0		0.2 ± 0.1		0.1 ± 0.0		0.0 ± 0.0	0.39	**0.0003**	0.39	0.09	0.79	0.73
** **															** **			** **		
**Mounting**	0.5 ± 0.1		0.4 ± 0.1		0.4 ± 0.1		0.3 ± 0.1	0.3 ± 0.1		0.2 ± 0.1		0.5 ± 0.1		0.3 ± 0.1	0.54	0.59	**0.01**	0.16	**0.03**	0.64

^1^Behaviours are expressed in frequencies per pig per hour observed. In case of continuous behaviour a new bout was scored when behaviour was > 30 seconds.

^2^LSC = low sanitary conditions, HSC = high sanitary conditions.

^3^LP = low dietary crude protein level, NP = normal dietary crude protein level.

^4^AA-B = basal dietary amino acid profile, AA-S = supplemented dietary amino acid profile containing 20% more Met, Thr, and Trp compared with the basal profile.

^5^*P*-values for manipulating other and belly nosing were based on square root transformed data. Significant *P*-values are indicated in bold and for a tendency values are underlined. Means are presented as raw means ± standard error.^6^n = number of pens, a pen contained nine pigs.

**Fig 2:**
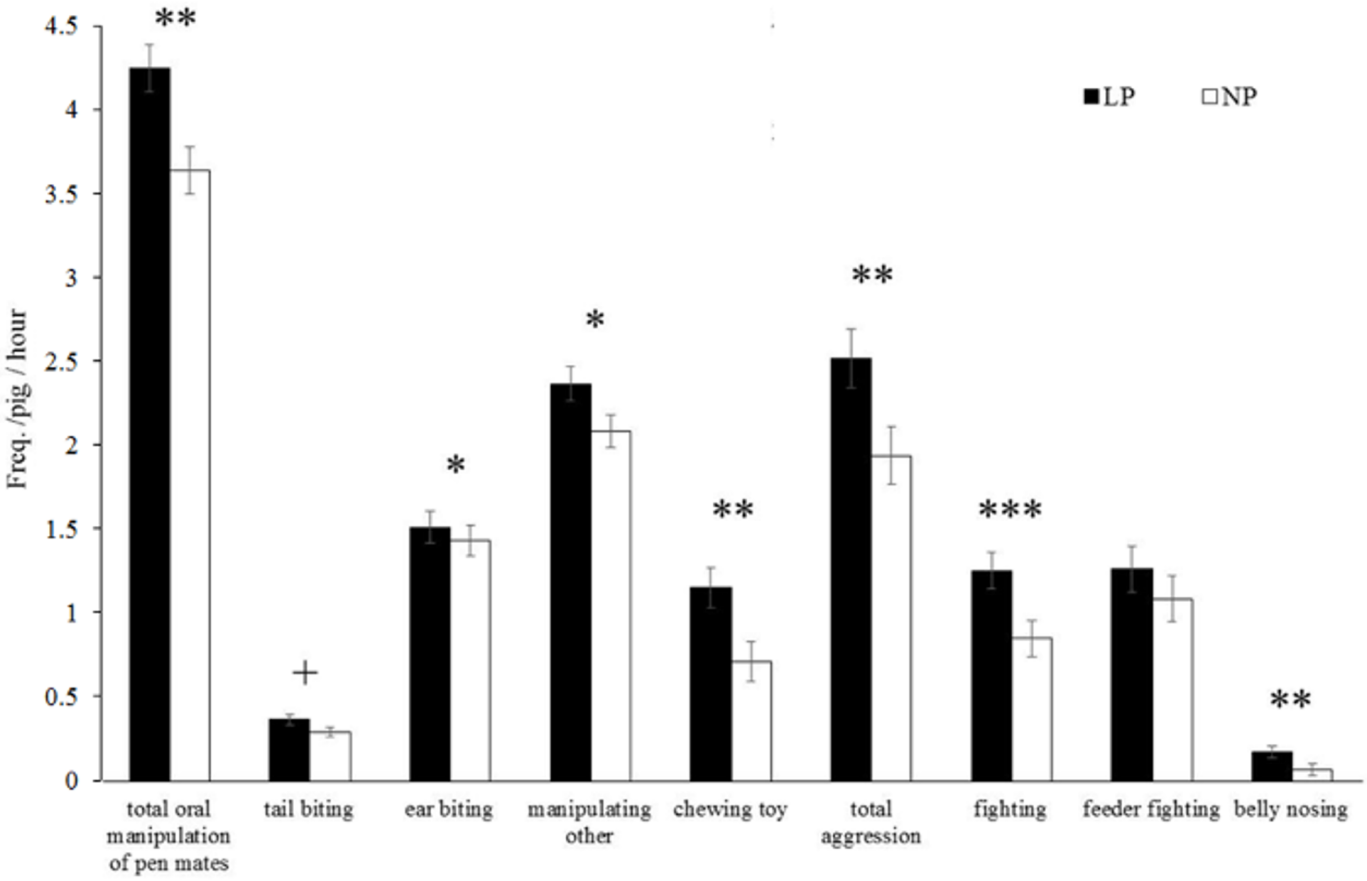
Behaviour of pigs during the grower and finisher phase for pigs fed diets with low (LP) or normal (NP) crude protein level. ^+^ *P* < 0.10, * *P* < 0.05, ** *P* < 0.01, *** *P* < 0.001.

Apart from sanitary conditions and dietary AA profile, also dietary protein level affected the pigs’ behaviour. The LP fed pigs showed higher frequencies of total oral manipulation of group mates (4.4 ± 0.4 vs. 3.8 ± 0.4 times per hour), ear biting (1.6 ± 0.2 vs. 1.3 ± 0.32 times per hour), manipulation of other body parts (2.5 ± 0.3 vs. 2.2 ± 0.3 times per hour), enrichment object biting (1.2 ± 0.2 vs. 0.8 ± 0.2 times per hour), total aggression (2.7 ± 0.3 vs. 2.1 ±0.3 times per hour), fighting (1.3 ± 0.2 vs. 0.9 ± 0.2 times per hour), belly nosing (0.2 ± 0.1 vs. 0.1 ± 0.0 times per hour) (All *P ≤* 0.05, Table 5, Fig 2), and a tendency for higher frequencies of tail biting than the NP fed pigs (0.4 ± 0.1 vs. 0.3 ± 0.1 times per hour; 0.05 ≤ *P ≤* 0.10).

Pigs fed with the AA-B diet had higher frequencies of mounting than pigs fed the AA-S diet (0.4 ± 0.1 vs. 0.3 ± 0.1 times per hour, *P* ≤ 0.05). Mounting was recorded more for LP than NP fed pigs in LSC (0.5 ± 0.1 vs. 0.4 ± 0.1 times per hour), but less for LP than NP fed pigs in HSC (0.3 ± 0.1 vs. 0.4 ± 0.1 times per hour) (sanitary conditions × dietary protein level interaction, *P* ≤ 0.05).

### Tail and ear damage scores

When considering the prevalence of tail damage as a binary score (tail damage vs. no tail damage), almost all of the pigs had tail lesions with a score higher than 1, i.e. at least bite marks, in at least one phase during the experiment. Furthermore, 39% of the pigs had a tail wound (score > 2), in at least one of the phases. These proportions were not affected by sanitary conditions or diet.

When averaging binary tail damage scores per pen and phase, the proportion of pigs with tail damage in LSC pens (0.80 ± 0.03) was higher than that in HSC pens (0.65 ± 0.03) in the starter phase (*P* ≤ 0.05; Table 6). In the grower phase, the proportion of pigs with tail damage in LSC (0.81 ± 0.04) was lower than that in HSC (0.92 ± 0.02; *P* ≤ 0.05, Table 6). An interaction for tail damage in the finisher phase was found for SC × CP, *P* ≤ 0.05; NP fed pigs in HSC (0.82 ± 0.04) had lower damage scores than LP fed pigs in HSC (0.92 ± 0.02), whereas this score was not different for pigs in LSC (0.91 ± 0.02 vs. 0.92 ± 0.02).

**Table 6:** Proportion of pigs (based on pen averages) with tail damage (score > 1) and ear damage (score > 1) observed for each treatment group: low and high sanitary condition pigs both with diets containing either low or normal protein levels and basal or supplemented amino acid profiles.

	LSC^1^				HSC^1^									
	LP^2^		NP^2^		LP		NP		*P-*values^4^					
Damage score^5^	AA-B^3^	AA-S^3^	AA-B	AA-S	AA-B	AA-S	AA-B	AA-S	SC	CP	AA	batch	SC×AA	SC×CP
**n**^**6**^	8	8	8	8	8	8	8	8						
**Tail**														
**starter**	0.80 ± 0.08	0.82 ± 0.04	0.76 ± 0.04	0.83 ± 0.08	0.64 ± 0.06	0.65 ± 0.06	0.78 ± 0.07	0.54 ± 0.04	**< .0001**	0.98	0.45	**0.002**	0.06	0.70
**grower**	0.83 ± 0.07	0.75 ± 0.09	0.78 ± 0.08	0.88 ± 0.06	0.95 ± 0.03	0.94 ± 0.02	0.94 ± 0.04	0.85 ± 0.06	**< .0001**	0.76	0.52	0.47	0.37	0.21
**finisher**	0.92 ± 0.03	0.91 ± 0.04	0.93 ± 0.03	0.91 x 0.04	0.95 ± 0.03	0.88 ± 0.03	0.81 ± 0.06	0.84 ± 0.04	0.68	0.07	0.54	**< .0001**	0.88	**0.04**
**Ear**														
**starter**	0.52 ± 0.05	0.41 ± 0.08	0.30 ± 0.07	0.45 ± 0.06	0.47 ± 0.12	0.36 ± 0.08	0.43 ± 0.06	0.28 ± 0.06	0.50	0.13	0.30	**0.02**	0.17	0.78
**grower**	0.34 ± 0.12	0.32 ± 0.06	0.37 ± 0.05	0.31 ± 0.07	0.42 ± 0.10	0.20 ± 0.05	0.34 ± 0.08	0.16 ± 0.05	0.10	0.63	**0.02**	0.86	0.11	0.53
**finisher**	0.53 ± 0.08	0.40 ± 0.06	0.51 ± 0.10	0.45 ± 0.09	0.31 ± 0.12	0.29 ± 0.05	0.32 ± 0.07	0.35 ± 0.12	**< .0001**	0.62	0.32	0.76	0.31	0.90

^1^LSC = low sanitary conditions, HSC = high sanitary conditions.

^2^LP = low crude protein level in diet, NP = normal crude protein level in diet.

^3^AA-B = basal dietary amino acid profile, AA-S = supplemented dietary amino acid profile containing 20% more Met, Thr, and Trp than the basal profile.

^4^ Significant *P*-values are indicated in bold and for a tendency values are underlined. Means are presented as raw means ± standard error of the mean.

^5^Damage scores were either 0 = no damage or 1 = damage and were averaged per pen.

^6^n = number of pens, a pen contained nine pigs.

In total, 78% of LSC and 58% of HSC pigs had at least in one phase an ear damage score higher than 1 (*P* ≤ 0.05). When averaging binary ear damage scores per pen and phase, the proportion of animals with ear damage was higher in pens fed AA-B diets (0.37 ± 0.04) compared to those fed AA-S diets (0.25 ± 0.03) in the grower phase (*P* ≤ 0.05). The proportion of pigs with ear damage in the grower phase tended to be higher for LSC (0.33 ± 0.04) than for HSC pigs (0.28 ± 0.04). For the finisher phase the proportion of ear damage was higher for LSC (0.92 ± 0.02) than for HSC pigs (0.87 ± 0.02; *P ≤* 0.05). The distribution of LSC and HSC pigs over tail and ear damage scores are presented in Tables B and C in S1 File, respectively.

When considering the prevalence of tail wounds as a binary score (tail wound vs. no tail wound), in LSC a lower proportion of pigs had tail wounds (0.13 ± 0.02) than in HSC (0.22 ± 0.03) in the grower phase (*P* ≤ 0.05; Table 7). The LP diet tended to result in a lower proportion of pigs with tail wounds (0.32 ± 0.04) compared with the NP diet (0.38 ± 0.04), in the grower phase (0.05 ≤ *P ≤* 0.10). Proportion of pigs with a tail wound tended to be lower for the AA-B diet (0.20 ± 0.03) than for the AA-S diet (0.14 ± 0.02) in the grower phase (0.05 ≤ *P ≤* 0.10). In the finisher phase, an interaction was found for SC × CP for proportion of pigs with tail wounds, in LSC a lower proportion of pigs with wounds was found when fed a LP diet (0.17 ± 0.03) instead of a NP diet (0.24 ± 0.03), whereas in HSC a higher proportion of pigs had tail wounds when fed a LP diet (0.22 ± 0.04) instead of a NP diet (0.15 ± 0.04) in the grower phase (*P* ≤ 0.05).

**Table 7:** Proportion of pigs (based on pen averages) with tail wounds (score > 2) and ear wounds (score > 3) observed for each treatment group: low and high sanitary condition pigs both with diets containing either low or normal protein levels and basal or supplemented amino acid profiles.

	LSC^1^				HSC^1^									
	LP^2^		NP^2^		LP		NP		*P-*values^4^					
Wound score^5^	AA-B^3^	AA-S^3^	AA-B	AA-S	AA-B	AA-S	AA-B	AA-S	SC	CP	AA	batch	SC×AA	SC×CP
**n**^**6**^	8	8	8	8	8	8	8	8						
**Tail**	** **													
**start**	0.08 ± 0.03	0.07 ± 0.03	0.11 ± 0.04	0.16 ± 0.05	0.07 ± 0.04	0.13 ± 0.05	0.13 ± 0.04	0.10 ± 0.03	0.10	0.17	0.50	0.43	0.99	0.38
**grower**	0.15 ± 0.04	0.04 ± 0.02	0.22 ± 0.06	0.10 ± 0.03	0.18 ± 0.06	0.21 ± 0.06	0.27 ± 0.08	0.22 ± 0.05	**0.007**	0.10	0.09	**0.0003**	0.14	0.80
**finish**	0.18 ± 0.03	0.15 ± 0.04	0.25 ± 0.05	0.23 ± 0.06	0.18 ± 0.06	0.26 ± 0.04	0.17 ± 0.06	0.12 ± 0.06	0.82	0.94	0.84	0.77	0.56	**0.04**
**Ear**														
**start**	0.05 ± 0.03	0.04 ± 0.04	0.00 ± 0	0.06 ± 0.03	0.06 ± 0.03	0.01 ± 0.01	0.00 ± 0	0.00 ± 0						
**grower**	0.05 ± 0.05	0.00 ± 0	0.00 ± 0	0.05 ± 0.03	0.17 ± 0.08	0.07 ± 0.03	0.00 ± 0	0.00 ± 0						
**finish**	0.06 ± 0.06	0.02 ± 0.02	0.00 ± 0	0.08 ± 0.04	0.08 ± 0.04	0.05 ± 0.02	0.00 ± 0	0.00 ± 0						

^1^LSC = low sanitary conditions, HSC = high sanitary conditions.

^2^LP = low crude protein level in diet, NP = normal crude protein level in diet.

^3^AA-B = basal dietary amino acid profile, AA-S = supplemented dietary amino acid profile containing 20% more Met, Thr, and Trp than the basal profile.

^4^ Significant *P*-values are indicated in bold and for a tendency values are underlined. Means are presented as raw means ± standard error of the mean. For ear wounds no statistically test was done, as there were too many pens without wounds.

^5^Wound scores were either 0 = no wound or 1 = wound and were averaged per pen.

^6^n = number of pens, a pen contained nine pigs.

When averaging binary ear wound scores (scores >3 for ear wounds vs. scores < 4 for) per pen and phase, many pens had a score 0 (no wound). Therefore no statistically test was performed on ear wound scores. The raw means are presented in Table 7.

## Discussion

In this study we assessed the influence of sanitary conditions, dietary protein concentration, and dietary AA profile on behaviour of pigs kept under practical conditions. To the best of our knowledge, this is the first study on damaging behaviour in pigs that are experimentally exposed to diverging sanitary conditions and receive different dietary treatments in a commercial farm setting. We demonstrate evidence for a causal relationship between sanitary conditions and damaging behaviour, indicating that biting behaviour, particularly ear biting in pigs is linked to health status in a diet-dependent manner. In addition, low dietary protein levels clearly increased the frequencies of both damaging biting behaviour and aggression. Dietary supplementation of Thr, Met and Trp modified these behavioural responses in low sanitary conditions pigs or low dietary protein fed pigs to some extent.

In our study ear biting and manipulation of body parts other than ears and tails were scored more frequently than tail biting. Given the frequent occurrence of tail wounds, tail docking was applied on the experimental farm. This is still common practice in the Netherlands and many other countries. It is known that ear biting occurs more frequently on farms with docked tails, likely because docked tails are not the most attractive body part to bite in [46]. Also in studies on pigs with intact [47] and half-docked tails [42], ear biting and manipulation of other body parts was observed more frequently than tail biting. Oral manipulation of pen mates occurred far more frequent than biting of the enrichment object provided, probably because body parts of conspecifics better meet the requirements of pigs for suitable chewing and rooting materials [48], and more pen mates than the enrichment objects which were available.

Tail docking, i.e. amputation of the tail or part of it in young pigs, is still done in several EU countries to prevent tail damage at later age [49]. The underlying causes of tail biting are, however, not removed by this practice, and tail wounds remain a problem [20]. Our results, with almost all of pigs showing at least bite marks and 39% having a small or large tail wound in one of the production phases, confirm that tail docking is no guarantee for the absence of tail biting. This is line with other studies. For instance, in a study with Irish slaughter pigs, 99% of the studied pigs were tail docked and 72.5% of these pigs had tail lesions at slaughter [50]. In an observation study with pigs with half-docked tails [42] frequency of ear biting (1.7 ± 0.1 per hour) in pigs was higher than tail biting (0.6 ± 0.1 per hour), as was the case in our study. Overall, the percentage of pigs with tail lesions was higher than the percentage of pigs with ear lesions, suggesting that, in spite of its relatively lower frequency, tail biting was more severe than ear damage in our study.

### Damaging behaviour and interactions with sanitary conditions and diet

Pigs in LSC showed more ear biting (+39%) compared with HSC pigs. Dietary supplementation with extra Met, Thr, and Trp reduced the frequency of ear biting (-16%) only in LSC pigs. The proportion of pigs with ear damage was higher in LSC than in HSC during the grower (tendency) and finisher phase. Similarly, proportions of pigs with tail damage was higher in LSC during the starter phase, although the opposite was found for proportion of pigs with tail damage or wounds in the grower phase. The LSC pigs had higher haptoglobin concentrations in blood and higher pleuritis scores for lungs at slaughter than HSC pigs [29], confirming a difference in health status. The impact of LSC on both damaging behaviours and the lesions and wounds they induce suggests a causal relationship between poor health and the occurrence of behavioural problems.

The increased propensity of pigs with a poor health status to start biting their pen mates could be related to increased immune system activation. In line with this, in laying hens the stimulation of immune reactivity, by intra-tracheal exposure to human serum albumin, increased damaging feather pecking [51], a redirected foraging behaviour showing a strong similarity to tail biting in pigs [52]. Immune stimulation may lead to a change in the animal’s nutrient requirements, particularly also for AA. Recent studies have shown indeed that the requirements for Trp [35, 36, 38], Met + cysteine, [37] and Thr [53, 54] are increased in case of immune system stimulation. Increased immune system activity in growing animals may redirect nutrients from growth to other processes that require AA. Indeed the LSC pigs in our study showed a lower body weight gain than the HSC pigs [29]. Notably, and unlike in most studies using model agents like LPS [37, 55, 56], Complete Freund’s Adjuvant (**CFA)** [38, 57], to stimulate the immune system, contrasts in sanitary status in this experiment occurred strictly without pigs showing signs of clinical illness. Pigs with suboptimal health may thus need more AA to support the immune system whilst maintaining growth. Indeed, particularly in the pigs kept under poor sanitary conditions, the diets with supplemented Met, Thr and Trp led to higher gain-to-feed ratios and a higher body weight gain [29]. If particular AA are limiting for optimal body functioning, pigs might intensify their chewing and rooting behaviour to satisfy their nutritional needs, and, under commercial conditions, direct part of their oral behaviours towards pen mates. This is confirmed by the lower frequency of ear biting recorded for AA-S compared with AA-B fed pigs in LSC. The frequency of tail biting and the ear and tail damage scores in LSC pigs were not always reduced by the supplementary AA diet. The former suggests that the link between health status, AA availability in metabolism, and damaging behaviour is complex and possibly influenced by multiple mechanisms.

An imbalance in AA might also influence behaviour by altering brain neurotransmitter metabolism. Several brain neurotransmitters are synthesized from AA [58, 59]. Threonine is a precursor of brain glycine [60], Met can be used as a donor of methyl groups used for synthesis of many substrates such as choline [61, 62], which is an acetylcholine precursor [63], and Trp is a precursor of serotonin (5-hydroxytryptamine; 5-HT) [62], a neurotransmitter which is known to affect behaviour and emotional state. Aggressive behaviour and stress have been shown to be related to the supply of dietary tryptophan and/or brain 5-HT in several animal and human studies [14–18], including studies on pigs [28, 64]. Ursinus et al. [19] recently found that pigs that perform tail biting behaviour had lowered blood 5-HT levels, and, in line with this, feather pecking chickens also showed altered peripheral [65] and central 5-HT [66, 67] metabolism. This could either be due to a direct effect of 5-HT on damaging behaviour demonstrated experimentally for chickens only [68], or reflect lowered AA availability for neurotransmitter synthesis, either or not caused by a lowered health status.

As Trp competes with other large neutral AA (**LNAA**) for passage over the blood brain barrier, the Trp: LNAA ratio in blood is a major determinant of brain 5-HT concentration [69].

Despite that there were differences in Trp: LNAA ratio between the AA-B and AA-S diets, no clear effects of dietary AA profile on aggression and on some of the damaging behaviours were found. In contrast, supplementation of AA in the AA-S diet resulted in lower recorded frequencies of mounting than for pigs fed the AA-B diet. It is not really clear via which mechanisms dietary AA provision affects mounting behaviour.

Apart from pigs in poor health being more likely to start biting, as indicated also affected by the dietary AA supply, the health-biting relationship [24, 70, 71] can be fortified by the poor ability of ill pigs to avoid being bitten [21]. Finally, it can be speculated that immune activation in wounded pigs (e.g. [25, 26], in turn, increases the probability of these pigs to start biting as well, which consequently leads to an escalation of tail or ear biting in the entire group.

### Dietary protein reduction: A risk for damaging behaviour?

Almost all damaging and aggressive behaviours recorded were more frequently scored for pigs fed the LP diet, considered to be dietary deficient in essential AA, than for those fed the NP diet, demonstrating a clear and direct effect of dietary protein level on damaging behaviour. In addition, a higher proportion of pigs with tail damage and tail wounds was found in HSC in the finisher phase when pigs were fed a LP diet. In a study of McIntyre and Edwards [12] pigs had a higher preference for a wet blood-soaked tail model when fed a low protein diet (98 g CP /kg) compared to their preference when fed a control diet (189 g CP/kg). These results suggest that a diet deficient in essential AA increases preference for blood. However, they used different protein sources in both diets [12]. Therefore an effect of protein source rather than CP level alone cannot be excluded in their study. In our study, however, the same protein sources at a different inclusion level were used in the respective diets. As average daily feed intake was not significantly different between LP an NP diets, we conclude that a the difference in AA uptake lead to more oral manipulation of pen mates and more aggression.

The LP diets might have led to a shortage or imbalance of AA resulting in restless pigs searching for missing nutrients. It might be that some brain neurotransmitters cannot be synthesized from AA derived from dietary protein as was stated by Harper and Peters [72], simply because insufficient precursors were available. Nutritional imbalance, probably due to an overall shortage in AA in this case, can increase foraging activity and redirected, damaging behaviours.

Apart from the effect on damaging behaviour, an overall shortage of the dietary supply of AA might also be responsible for the increase in aggressive behaviour in pigs fed the LP diets. The LP diets, regardless of the AA profile, were diets with a lower Trp concentration compared with the NP diets. A low Trp concentration in the brain, due to a low concentration of Trp in the diet, might affect brain serotonin metabolism resulting in more aggressive behaviour as found by Martinez-Trejo et al. [13], or might affect aggressive behaviour via other hormones such as cortisol or noradrenaline as found by Koopmans et al. [64]. Also the effect of AA concentration on blood insulin concentration might have played a role, as blood insulin is known to play a role in Trp uptake in the brain as well [70].

Alternatively, Fraser et al. [7] suggested that the effect of deficient dietary essential AA level on biting behaviour is indirectly caused by a lower growth rate of these animals, rather than by the dietary composition as such. In support of this, Mc Intyre and Edwards [12] found a tendency for a correlation between body weight gain and preference for a blood tail model vs. water tail model. Also Larsen [73] found that tail biting pigs often were slower growing animals. In contrast, Ursinus et al. [42] reported a higher phenotypic and genotypic growth in fanatic tail biters. These authors suggested that fast growing pigs and pigs with a low growth rate due to health problems may have a high metabolic demand for protein synthesis for muscle tissue or immune processes in common, which could explain their increased biting tendencies. A larger variation in size of animals within the same pen has also been reported to negatively influence biting behaviour [21]. When calculating the standard error for body weight per pen in our study the standard error was higher for LSC than HSC animals, which means that variation in weight per pen was higher in LSC animals. When calculating this standard error for body weight with the measured behaviours, no clear correlations were found.

Even if growth rate is related to biting behaviour, more research is needed to determine cause and effect, and to study the impact of a potential shared underlying factor, such as, for instance, an actual status or history of poor health. A low protein diet with deficiencies in essential AA should thus be considered as a risk factor for biting behaviour and aggression in pigs, which can only partly be counteracted by supplementation with specific essential AA, such as Met, Thr and Trp.

In conclusion, this study shows a causal link between sanitary conditions, dietary CP level and damaging behaviours. Both LSC and a reduction of dietary CP level, increase the occurrence of damaging behaviours in pigs and therefore may negatively impact pig welfare. These effects could only partly be counteracted by supplementation with specific essential AA that are known to be increasingly required in case of immune system activation.

Tail and ear biting clearly are multifactorial problems and combating these requires a multidisciplinary effort. This study demonstrates that care should be taken in reducing dietary protein concentrations to improve protein efficiency in pigs, even when maintaining the ratio of the most important essential AA relative to energy. It incurs a risk to increased damaging behaviours, particularly when pigs are kept under poor sanitary conditions.

## Supporting information

S1 File **Table A. Formulated dietary amino acid profiles relative to lysine on apparent ileal digestible level, fed in combination with different dietary protein levels to pigs kept under different sanitary conditions.** ^1^ AA-B = basal dietary amino acid profile; AA-S profile = supplemented dietary amino acid profile with 20% extra Met, Thr, and Trp than basal profile. **Table B. The percentage of pigs in different tail damage score categories from observations taken across all phases for low and high sanitary conditions.** Score 1: No tail damage; 2: Bite marks; 3: Small wound; 4: Medium wound, part of tail missing; and 5: Severe wound, no tail is left. LSC: low sanitary condition pigs, HSC: high sanitary condition pigs. **Table C. The percentage of pigs in different ear damage score categories from observations taken across all phases for low and high sanitary conditions.** Score 1: No ear damage, 2: Top or bottom lesions, 3: Top and bottom lesions, 4: Severe damage, part of the ear is missing, and 5: ear necrosis. LSC: low sanitary condition pigs, HSC: high sanitary condition pigs.
(DOCX)
