# Supplementary material for: A link between damaging behaviour in pigs, sanitary conditions, and dietary protein and amino acid supply
Source: PLoS One. 2017 May 8;12(5):e0174688. doi: 10.1371/journal.pone.0174688 (PMC5421778; doi:10.1371/journal.pone.0174688)
Supplement: S1 File — Table A. Formulated dietary amino acid profiles relative to lysine on apparent ileal digestible level, fed in combination with different dietary protein levels to pigs kept under different sanitary conditions. 1 AA-B = basal dietary amino acid profile; AA-S profile = supplemented dietary amino acid profile with 20% extra Met, Thr, and Trp than basal profile. Table B. The percentage of pigs in different tail damage score categories from observations taken across all phases for low and high sanitary conditions. Score 1: No tail damage; 2: Bite marks; 3: Small wound; 4: Medium wound, part of tail missing; and 5: Severe wound, no tail is left. LSC: low sanitary condition pigs, HSC: high sanitary condition pigs. Table C. The percentage of pigs in different ear damage score categories from observations taken across all phases for low and high sanitary conditions. Score 1: No ear damage, 2: Top or bottom lesions, 3: Top and bottom lesions, 4: Severe damage, part of the ear is missing, and 5: ear necrosis. LSC: low sanitary condition pigs, HSC: high sanitary condition pigs. (DOCX) [file pone.0174688.s001.docx]

**S1 supporting information**

S1 File. Table A) Formulated dietary amino acid profiles relative to lysine on apparent ileal digestible level, fed in combination with different dietary protein levels to pigs kept under different sanitary conditions. ^1^ AA-B = basal dietary amino acid profile ; AA-S profile = supplemented dietary amino acid profile with 20% extra Met, Thr, and Trp than basal profile. Table B) The percentage of pigs in different tail damage score categories from observations taken across all phases for low and high sanitary conditions. Score 1: No tail damage; 2: Bite marks; 3: Small wound; 4: Medium wound, part of tail missing; and 5: Severe wound, no tail is left. LSC: low sanitary condition pigs, HSC: high sanitary condition pigs. Table C) The percentage of pigs in different ear damage score categories from observations taken across all phases for low and high sanitary conditions Score 1: No ear damage, 2: Top or bottom lesions, 3: Top and bottom lesions, 4: Severe damage, part of the ear is missing, and 5: ear necrosis.

LSC: low sanitary condition pigs, HSC: high sanitary condition pigs.

A.

| **Amino acid** | **AA-B profile**^1^ |  | **AA-S profile**^1^ |
| --- | --- | --- | --- |
| Lysine | 100 |  | 100 |
| Methionine + cysteine | 51 |  | 61 |
| Threonine | 59 |  | 71 |
| Tryptophan | 18 |  | 22 |
| Arginine | 87 |  | 87 |
| Histidine | 43 |  | 43 |
| Isoleucine | 53 |  | 53 |
| Leucine | 101 |  | 101 |
| Phenylalanine | 54 |  | 54 |
| Valine | 69 |  | 69 |

B.

| **Score** | **LSC** | **HSC** |
| --- | --- | --- |
| **1** | 15.6 | 18.5 |
| **2** | 69.4 | 64.6 |
| **3** | 14.5 | 16.5 |
| **4** | 0.5 | 0.5 |
| **5** | 0.0 | 0.0 |

C.

| **Score** | **LSC** | **HSC** |
| --- | --- | --- |
| **1** | 73.4 | 77.4 |
| **2** | 20.8 | 15.6 |
| **3** | 1.3 | 1.4 |
| **4** | 0.1 | 0.8 |
| **5** | 4.4 | 4.7 |
